# miR-24 Regulates Macrophage Polarization and Plasticity

**DOI:** 10.4172/2155-9899.1000362

**Published:** 2015-10-28

**Authors:** Jezrom B. Fordham, Afsar R. Naqvi, Salvador Nares

**Affiliations:** Department of Periodontics, University of Illinois at Chicago, Chicago, Illinois, USA

**Keywords:** Macrophages, microRNA, Polarization, Plasticity, Periodontitis

## Abstract

**Objective:**

MicroRNAs (miRNA) are ubiquitous regulators of human biology and immunity. Previously, we have demonstrated an inhibitory role for miR-24 in the phagocytosis of *Escherichia coli* and *Staphylococcus aureus* bioparticles and the induction of cytokine secretion in response to lipopolysaccharide (LPS) of the same origin; also, we have identified divergent and convergent miRNA responses to LPS from the periodontopathic pathogens *Aggregatibacter actinomycetemcomitams* (Aa) and *Porphyromonas gingivalis* (Pg), and revealed cigarette smoke extract as an environmental modifier of Pg LPS structure (Pg CSE) impacting macrophage miRNA responses. This study was designed to investigate the role of miR-24 on macrophage polarization and plasticity.

**Methods:**

Primary human macrophages were differentiated from CD14^+^ monocytes isolated from peripheral blood mononuclear cells (PBMCs) by MACS positive selection and transfected with miR-24 miRNA mimics, inhibitors, or negative control mimic; followed by stimulation with cytokines and/or LPS under various conditions representing key stages of macrophage activation. Macrophage activation and polarization was assessed using assays for cytokine production (ELISA) and protein expression (flow cytometry, immunoblot). MiR-24 expression was assessed by RT-PCR.

**Results:**

Stimulation of macrophages with LPSs of Aa, Pg, and Pg CSE origin resulted in dissimilar levels of cytokine expression and differential expression of miR-24. Overexpression of miR-24 inhibited cytokine secretion in response to LPS. Priming of macrophages with interferon gamma (IFN-γ) did not overcome this inhibitory effect, but classical activation of macrophages with IFN-γ plus TNF-α, TNF-β, or IL-17, modulated the pattern of miR-24 mediated suppression in a cytokine-specific fashion. Overexpression of miR-24 enhanced CD206 upregulation during alternative macrophage activation and inhibited its downregulation in macrophage transitioning from alternative to classical activation states. Overexpression of miR-24 resulted in reduced expression of the Class 1A PI 3-kinase subunit p110 delta (p110δ).

**Conclusion:**

Pathogen- and environment-specific modifications in LPS alter the expression of cytokines and miR-24 in human macrophages. MiR-24 is a negative regulator of macrophage classical activation by LPS and promotes alternative activation under conditions of polarization and plasticity. MiR-24 mediated inhibition of LPS-induced cytokine secretion is dependent upon macrophage activation state at the point of stimulation, and this may be due to the degree to which p110δ is involved in the intracellular signaling pathway/s that transduce receptor ligation into cytokine induction. While important differences were observed in the effect of miR-24 on macrophages, these data indicate that overexpression of miR-24 would be predominantly anti-inflammatory

## Introduction

Macrophage (Mφ) activation can occur via the recognition of pathogen-associated molecular patterns (PAMPs) by pathogen-recognition receptors (PRRs, including the toll-like receptors [TLRs]) or by cytokine-receptor ligation, with nuclear factor- kappa B (NF-κB) activation being a common downstream effect of these stimuli [1]. Interferon gamma (IFN-γ) and tumour necrosis factor alpha (TNF-α) are the most potent inducers of classical Mφ activation and production of these cytokines is the major route by which T helper (Th) subset 1 (Th1) cells enhance the antimicrobial capacity of Mφ [2]. Interleukin (IL)-4 (IL-4) and IL-13 are the most potent inducers of alternative Mφ activation, and the production of these cytokines by Th2 cells is central to the establishment of the wound-healing state that accompanies the resolution of the inflammatory state and the catabasis of adaptive immune responses [2].

The concept of Mφ plasticity includes two key points: 1) Mφ activation can result in a spectrum of effector functions (and expression of phenotypic markers) that are informed by environmental, immunological and pathogen-specific factors, and 2), Mφ respond dynamically to these signals to rapidly, and specifically alter one or more of their present functional parameters. Mφ activation is a central component of the immunopathology that occurs in periodontitis, an infectious, inflammatory disease. Etiological factors include the gram-negative anaerobes, *Aggregatibacter actinomycetemcomitams* (Aa) [3] and *Porphyromonas gingivalis* (Pg) [4], while the smoking of cigarettes is considered a significant risk factor [5]. Further, cigarette smoke has been shown to modify the structure of the LPS produced by Pg resulting in altered leukocyte responses [6].

A large proportion of reports published on miRNA expression in Mφ have concentrated on PRRs- particularly the TLRs; while relatively few have identified miRNAs that modulate Mφ activation and plasticity meted by other stimuli. The TLRs and their associated signaling molecules have proven to be rich targets for miRNA regulation, and include miR-155, miR-21 and miR-146a targeting of TLR4 signaling [7,8], miR-24 targeting of MD-1 [9], miR-9 and 125b targeting of the TLR4/IL-1R signaling components IRAK-1, TRAF6, IKKe and p50NF-jB [10,11]; miR-17/20a/106a targeting of signal-regulatory protein α (SIRPα) [12], and miR-98 regulation of IL-10 production [13]. While not exhaustive, this list alone establishes miRNA as a regulator of Mφ phenotype.

Our previous bioinformatic analysis of miR-24 identified numerous predicted targets with roles in intracellular signaling pathways known to be central to Mφ activation and polarization, including members of the PI-3 kinase family [9]. Three classes of PI 3-kinases exist: Class I, II and III; and of these it is Class I that appear to be most actively involved in immune responses [14]. Class I PI 3-kinases are heteromeric and are composed of a regulatory subunit combined with a 110 kDA catalytic subunit (Class 1A=p110α, β or δ; Class 1B=p110γ). Of these catalytic subunits, p110δ is the most closely associated with leukocyte development and function [14]. All 3 classes of PI 3-kinases function in a similar fashion: in response to receptor activation (including cytokine/chemokines, TLRs and TCR/BCR) they generate phosphatidylinositol 3,4,5-trisphosphate (PIP3) and PIP3 recruits receptor tyrosine kinases, such as PDPK1 and its main effector, AKT1 (Protein kinase B).

The recent recognition of miRNA (and other small, non-coding, regulatory RNA-based molecules) as a rapidly induced mechanism of transient regulation of gene expression has provided both an explanation and a means to manipulate Mφ polarization. The myeloid arm of the immune system, including Mφ and their precursor monocytes, are in many ways ideal candidates for miRNA-based therapy. There is also the continued perception of miRNA as ‘fine-tuners’ of gene expression- a notion that runs contrary to most therapeutic approaches where high impact with low toxicity seems to be the prevailing goal. Indeed, miRNA has highlighted how small changes in specific gene expression can have profound effects on biological processes. Here we show that miR-24 is a negative regulator of Mφ classical activation by LPS and promotes alternative activation under conditions of polarization and plasticity.

## Materials and Methods

### Primary human monocyte isolation and Mφ differentiation

Freshly prepared buffy coats were collected from healthy human donors (n=3–4) (Sylvan N. Goldman Oklahoma Blood Institute, Oklahoma City, OK, USA) and CD14^+^ monocytes obtained by density gradient centrifugation and magnetic bead isolation, as previously described [9]. Briefly, PMBCs were purified using Ficoll Paque™ (GE Healthcare, Piscataway, NJ, USA) based density centrifugation. PBMCs were incubated with magnetically labeled CD14 beads (Miltenyi Biotech, Cologne, Germany) according to manufacturer’s instructions. Monocyte purity and viability was >95%, as determined by flow cytometry [9]. For mD-Mφ differentiation, monocytes were plated at a density of 2×10^6^/ml in DMEM supplemented with penicillin (100 U/ml), streptomycin (100 µg/ml) and gentamicin (50 µg/ml). After 2 hours the media was substituted with media containing 10% heat-inactivated FBS (Life Technologies, Grand Island, NY, USA), and rhM-CSF (50 ng/ml; Peprotech, Rocky Hill, NJ, USA). Media was replaced every 72 h. At day 7, cells were harvested and differentiation confirmed by flow cytometric analysis. Mφ were utilized between days 7–10.

### LPS preparation

*Porphyromonas gingivalis* strain W83 was grown in the absence or presence of cigarette smoke extract, as described previously [15]. In brief, *Porphyromonas gingivalis* cultures were grown to mid to late exponential phase in Gifu Anaerobe Medium (GAM, Nissui Pharmaceutical, Tokyo, Japan) or GAM-CSE under anaerobic conditions at 37°C in a Coy Laboratories anaerobic chamber (Coy Laboratory Products, Grass Lake, MI, USA). For Pg-CSE cultures, GAM was conditioned using standard reference cigarettes (Kentucky Tobacco Research and Development Centre, Lexington, KY, USA) and diluted to 4000 ng/ml nicotine equivalents prior to use [16,17]. LPS extraction kit (iNtron Biotechnology, Kyunggi-do, Korea) was used for LPS extraction. LPS from *Aggregatibacter actinomycetemcomitans* strain Y4 (serotype B) was extracted and purified as previously described [18]. *Escherichia coli* (0111B4) LPS was purchased from Invitrogen (Invitrogen, Carlsbad, CA, USA) and included as an example of a non-periodontopathic bacterium for comparison. LPS was found to contain <0.001% nucleic acid and 0.7% protein by spectrophotometry and bicinchoninic acid protein assay, respectively [18].

### Total RNA isolation

Total RNA was isolated using the miRNeasy kit (Qiagen, Germantown, MD, USA), following the manufactures’ guidelines and assessed using the NanoDrop (ThermoScientific, Wilmington, DE, USA).

### Quantitative real-time PCR

miRNA expression was assayed using miScript primers and the miScript II RT kit (both from Qiagen) as previously described [9,19]. One hundred fifty nanograms of total RNA was reverse transcribed and reactions were run using miRNA specific primers, universal primer, and PCR mix buffer. Expression was normalized to RNU6B (U6) expression. Ct values of replicates were analyzed to calculate relative fold change using the delta-delta Ct method [20].

### Bioinformatic analysis

Bioinformatic analysis of predicted miRNA targets was performed using miRWalk (http://www.umm.uni-heidelberg.de/apps/zmf/mirwalk/mirnatargetpub.html) [21] as we previously described [9,19]; targets identified by 3 or more of the 8 predictive algorithms were considered as putative targets.

### Transient miRNA transfections

MiScript miRNA mimics and inhibitors were purchased from Qiagen. AllStars negative mimics (Qiagen) were used as controls. Transient transfections were performed using Lipofectamine 2000 (Life Technologies) according to manufacturer’s instructions. Day 7 differentiated mD-Mφ were transfected at a final concentration of 50 nM. Red siGLO oligos (Thermo Fisher Scientific, Waltham, MA, USA) were used to confirm successful transfection, with transfection efficiency being greater than 90% as previously reported [9]. Cell viability was assessed using the CellTiter 96 AQueous Cell Proliferation Assay Kit (Promega, Madison, WI, USA). Viability of all cell culture samples was analyzed 2 hours prior to harvesting of cells/supernatant for subsequent experimental analysis.

### Stimulation and activation

Cells were stimulated with LPS (Ec, Aa, Pg, Pg CSE) at a concentration of 100 ng/ml. Recombinant human IFN-γ, TNF-α, TNF-β, IL-17A, IL-4 and IL-13 were purchased from Peprotech (Peprotech, Rocky Hill, NJ, USA). Cytokines were titrated prior to use with effective concentrations selected based upon their ability to alter LPS (Ec) induced concentrations of pro-/anti-inflammatory cytokines as measured by ELISA. Concentrations used for experiments were: IFN-γ, 100 ng/ml; TNF-α, 20 ng/ml; TNF-β, 20 ng/ml; IL-17A, 100 ng/ml; IL-4, 100 ng/ml; IL-13, 10 ng/ml. Treatment of mD-Mφ with these cytokines and concentrations had no significant effect on viability after 72 h.

### Flow cytometry

The expression of surface markers was analyzed using Abs specific for human CD206, CD163, and CD45 (all purchased from BD Pharmingen, San Jose, CA, USA). Corresponding fluorescence label-conjugated isotype controls were utilized in all experiments. In brief, cells were washed once in room temperature flow buffer (PBS supplemented with 1% (v/v) FBS and 0.05% NaN_3_), once in ice-cold flow buffer and cell staining was performed on ice. Samples were analyzed using a BD Cyan flow cytometer using CellQuest software (BD Biosciences, San Jose, CA, USA). Further analysis was performed using FloJo software (Tree Star Inc., Ashland, OR, USA).

### Enzyme-linked immunosorbent assay (ELISA)

Human capture and detection Abs (Life Technologies) were used for the development of TNF-α, IL-12p40, IL-6 and IL-10 sandwich ELISA according to the manufacturer’s instructions. Cell culture supernatants were analyzed using a 96-well plate reader with absorbance at 450 nm (reference 650 nm).

### Western blot

Cells were lysed in cell lysis buffer (Cell Signaling Technology, Davers, MA, USA) supplemented with protease inhibitors (Roche, Basel, Switzerland). Protein content was estimated using the Bradford assay (BioRad Laboratories, Hercules, CA, USA). Equal amounts of protein were resolved in 10% Mini-PROTEAN^®^ TGX™ (BioRad) gels and electrotransferred to nitrocellulose membranes (GE Healthcare). Membranes were blocked with 5% skimmed milk for 2 hours and incubated with primary antibodies against p110δ or GAPDH (1:1000; Cell Signaling Technologies) overnight at 4°C. The blots were washed with PBS 0.1% Tween 20 thrice before incubating with secondary antibody (1:10,000) for 1 hour at room temperature. Blots were washed with PBS 0.1% Tween 20 thrice and protein detection was performed using enhanced chemiluminescence (GE Healthcare). ImageJ software (http://rsbweb.nih.gov/ij/) was used to quantify the results. The values for each lane were normalized with respect to endogenous control GAPDH.

### Statistical analysis

Data was analyzed for statistical significance using GraphPad Prism GraphPad Software, La Jolla, CA, USA). P-values were calculated using either Student’s t-test or by ANOVA.

## Results

### Stimulation of Mφ with LPS from periodontal pathogens results in convergent and divergent cytokine and miRNA expression

**Figure 1:**
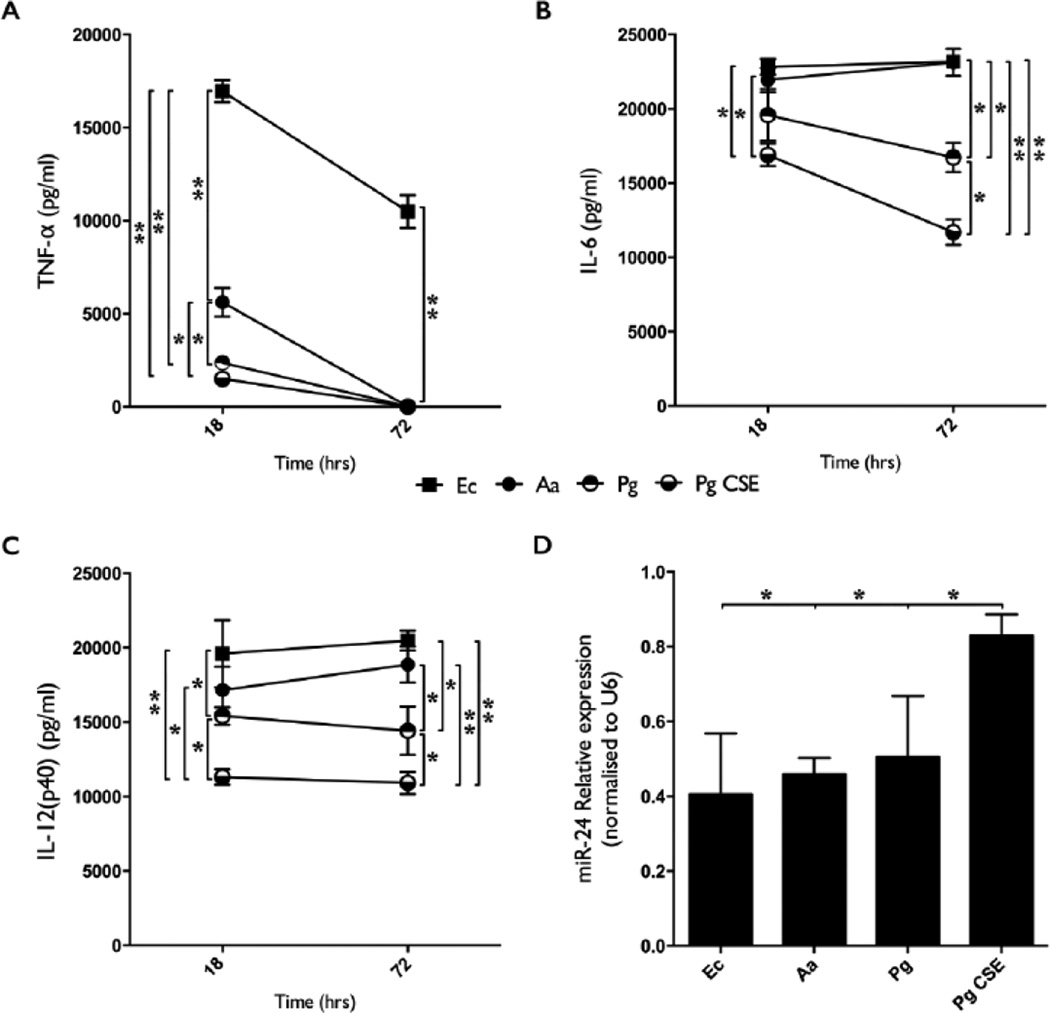
Aa-, Pg, and Pg CSE-derived LPS display differing capacities to induce TNF-α, IL-6 and IL-12(p40) secretion and miR-24 downregulation in macrophages. **A)** TNF-α levels in the culture supernatant of macrophages stimulated with Aa-, Pg-, Pg CSE-, or Ec-LPS (all at 100 ng/ml) was assayed by ELISA after 18 and 72 h of stimulation. **B)** IL-6 levels assayed by ELISA. **C)** IL-12(p40) levels assayed by ELISA. **D)** miR-24 expression was assessed by RT-PCR after 18 h of stimulation. A, B, C=Mean values, error bars (standard deviation), and significance calculated by ANOVA). n=4 biological donors. D=Mean values, error bars (standard deviation), and significance calculated using Student’s t-test (two-tailed). n=3 biological donors. (^*^P<0.05, ^**^P<0.001).

Mature (day 7) mD-Mφ were stimulated with LPS (100 ng/ml) derived from Aa, Pg, Pg CSE or Ec. Supernatant levels of TNF-α, IL-6 and IL-12p40 were measured by ELISA at 18 and 72 hours post stimulation (Figures 1A–1C). LPS from periodontal pathogens (Aa, Pg, Pg CSE) induced less TNF-α production than Ec-LPS, and Aa-LPS induced higher levels of TNF-α than either Pg- or Pg CSE-LPS at 18 hours (Figure 1A). In contrast to Ec-LPS stimulation, none of the periodontopathic-derived LPS’ resulted in detectable levels of TNF-α at the 72 h time-point.

After 18 hours the levels of IL-6 present were comparable between Aa-, Pg-, and Ec-LPS stimulated cultures, but levels in Pg CSE-LPS stimulated cultures were significantly lower. At the 72 h time-point, IL-6 levels were lower with Pg- or Pg CSE-LPS compared to Aa- or Ec-LPS, while IL-6 levels in Pg CSE-LPS stimulated cultures was significantly lower than in Pg-LPS stimulated cultures (Figure 1B). Aa-LPS stimulation resulted in IL-12p40 levels that were comparable to those of Ec-LPS stimulation at both time-points tested; however, significant differences between Aa- and Pg-/Pg CSE-LPS, as well as between Pg- and Pg CSE-LPS were present at both time-points (Figure 1C).

miR-24 expression at the 18 hour time-point was analyzed by RT-PCR (Figure 1D). Stimulation with Ec-, Aa- or Pg-LPS, but not Pg CSE-LPS, significantly downregulated miR-24 expression relative to unstimulated control cultures.

### miR-24 inhibits the production of pro-inflammatory cytokine production by Mφ in response to LPS from periodontal pathogens

**Figure 2:**
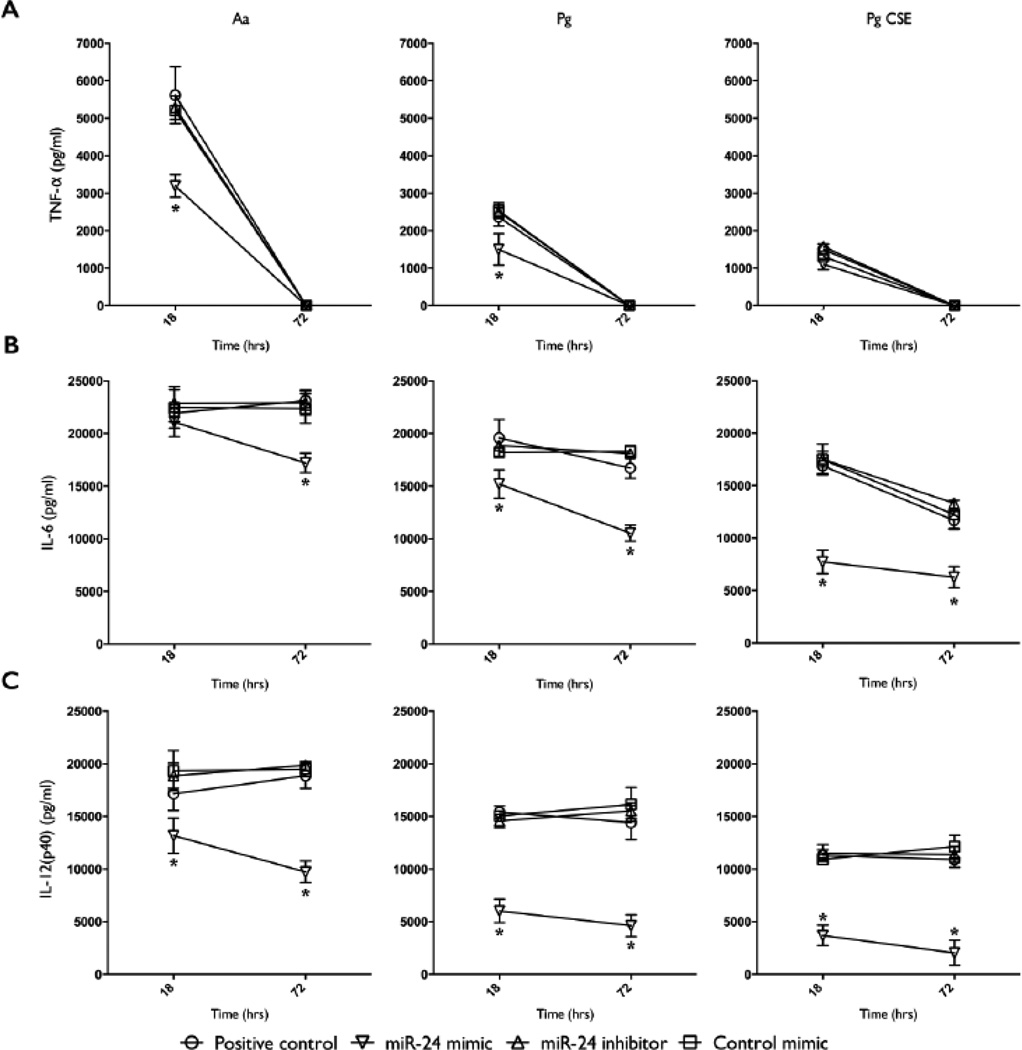
Overexpression of miR-24 inhibits the secretion of TNF-α, IL-6 and IL-12(p40) (measured by ELISA) by macrophages stimulated with Aa-, Pg-, and Pg CSE-derived LPS. **A)** TNF-α levels in the culture supernatant of macrophages transfected with miR-24 mimic, inhibitor, or control mimic (all at 50 nM), or positive control (untransfected) for 18 h, and stimulated with Aa-, Pg-, or Pg CSE-LPS (all at 100 ng/ml) was assayed by ELISA at 18 and 72 h of stimulation. **B)** IL-6 levels assayed by ELISA. **C)** IL-12(p40) levels assayed by ELISA. A, B, C = Mean values, error bars (standard deviation), and significance calculated by ANOVA). n=4 biological donors. (^*^P<0.05, ^**^P<0.001).

Mature mD-Mφ were transfected with miR-24 mimic, inhibitor, or negative control mimic, for 18 hours, and stimulated with LPS and levels of TNF-α, IL-6 and IL-12p40 were measured by ELISA at 18 and 72 hours post stimulation. Compared to cultures transfected with miR-24 inhibitor or control mimic, or positive control (untransfected), transfection with miR-24 mimic resulted in lower TNF-α levels at the 18 hour time-point in cultures stimulated with Aa-LPS or Pg-LPS (Figure 2A). This effect was not observed with Pg CSE-LPS. TNF-α was not detected at the 72 hour time-point in any of the culture supernatants. Similarly, transfection with miR-24 mimic resulted in lower levels of IL-6 at both time-points in Pg-LPS and Pg CSE-LPS stimulated cultures, but only at the 72 hour time-point in Aa-LPS stimulated cultures (Figure 2B). Transfection with miR-24 mimic, but not inhibitor nor control mimic, resulted in lower levels of IL-12p40 at both time-points in cultures stimulated with Aa-, Pg- or Pg CSE-LPS (Figure 2C).

### miR-24-mediated inhibition of LPS induced cytokine production is dependent upon Mφ activation state

**Figure 3:**
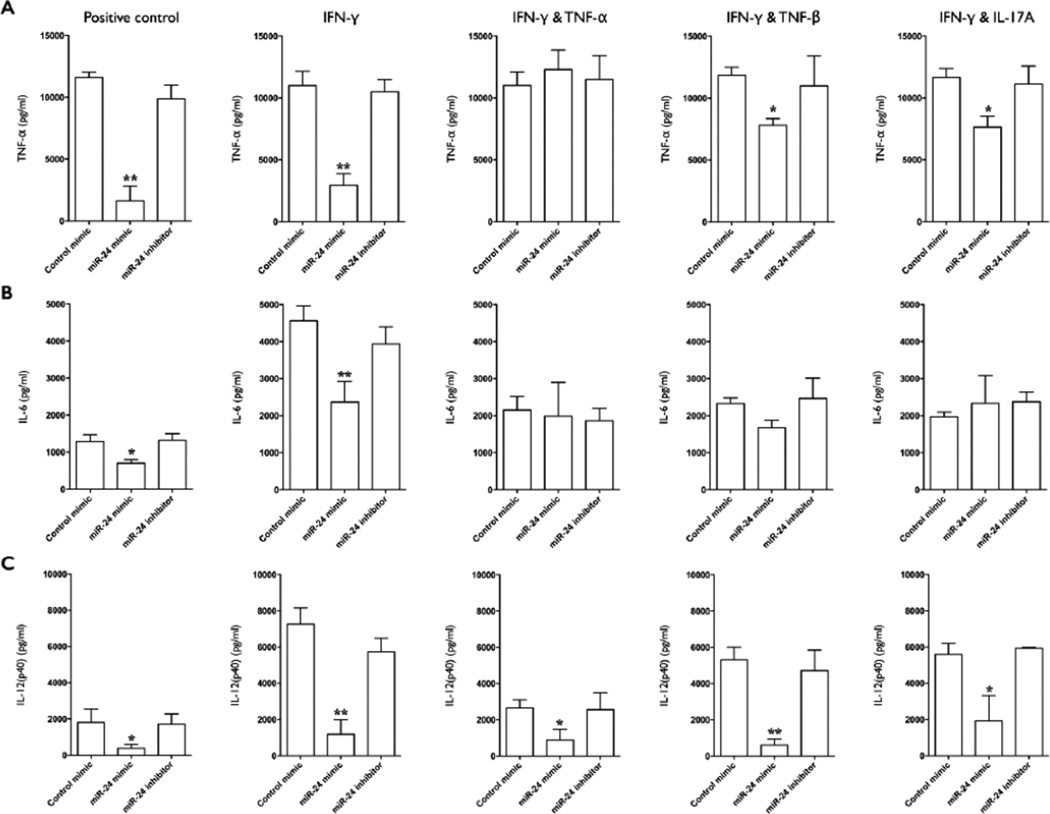
Macrophage activation state modulates the inhibitory capacity of miR-24 on Aa-LPS induced cytokine secretion. **A)** TNF-α levels in the culture supernatant of macrophages transfected with miR-24 mimic, inhibitor, control mimic (all at 50 nM), or positive control (untransfected) for 18 h, primed with IFN-γ (10 ng/ml), IFN-γ plus TNF-α (1 ng/ml), IFN-γ+TNF-β (1 ng/ml), IFN-γ plus IL-17A (10 ng/ml), or positive control (no cytokine priming) for 18 h, and stimulated with Aa-LPS (100 ng/ml) for 12 h, was assayed by ELISA. **B)** IL-6 levels assayed by ELISA. **C)** IL-12(p40) levels assayed by ELISA. A, B, C=Mean values, error bars (standard deviation), and significance calculated by ANOVA). n=4 biological donors. (^*^P<0.05, ^**^P<0.001).

Mature mD-Mφ were sequentially transfected with miR-24 mimic, inhibitor, or negative control mimic for 18 hours, then treated with cytokines that promote classical Mφ activation (IFN-γ, IFN-γ and TNF-α, IFN-γ and TNF-β, IFN-γ and IL-17A) for 18 h, and then culture media was replaced with fresh media containing Aa LPS. Levels of TNF-α, IL-6, IL-12(p40) in the supernatant at 12 hours post stimulation was measured by ELISA (Figure 3). Transfection with miR-24 mimic, but not inhibitor or control mimic, inhibited TNF-α production in positive control (no cytokine pre-treatment) and cultures pre-treated with IFN-γ, IFN-γ and TNF-β, and IFN-γ and IL-17A, but TNF-α production was restored to untransfected levels by IFN-γ and TNF-α pre-treatment (Figure 3A). Pre-treatment with IFN-γ and TNF-β or IFN-γ and IL-17A resulted in levels of TNF-α in miR-24 mimic transfected cultures that were intermediate to those of IFN-γ and IFN-γ and TNF-α.

**Figure 4:**
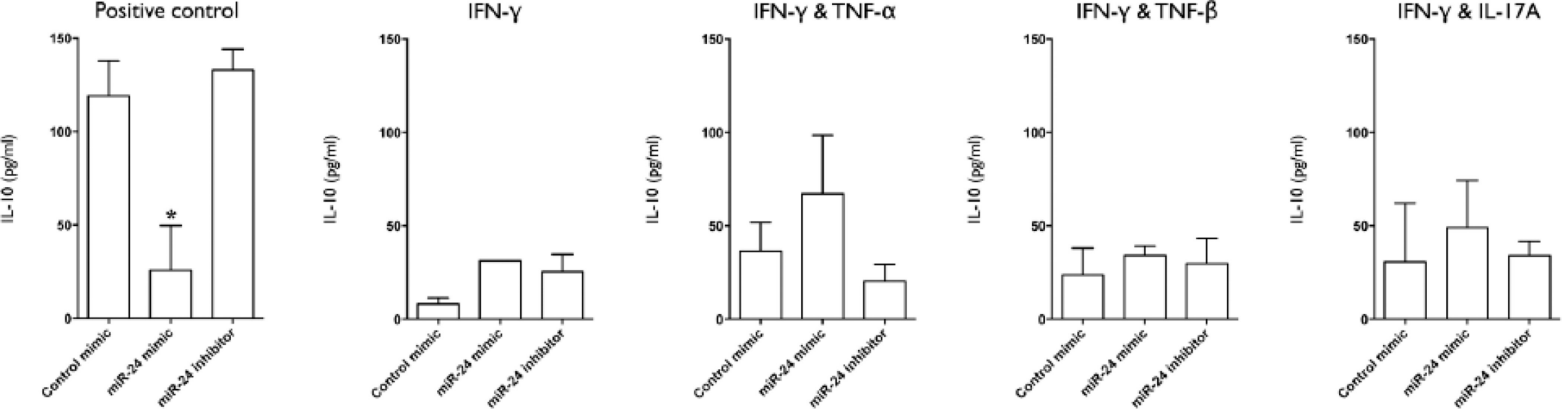
Overexpression of miR-24 inhibits early Aa-LPS induced IL-10 production in resting but not classically primed macrophages. IL-10 levels in the culture supernatant of macrophages transfected with miR-24 mimic, inhibitor, control mimic (all at 50 nM), or positive control (untransfected) for 18 h, primed with IFN-γ (10 ng/ml), IFN-γ plus TNF-α (1 ng/ml), IFN-γ+TNF-β (1 ng/ml), IFN-γ plus IL-17A (10 ng/ml), or positive control (no cytokine priming) for 18 h, and stimulated with Aa-LPS (100 ng/ml) for 96 h, was assayed by ELISA. A, B, C=Mean values, error bars (standard deviation), and significance calculated by ANOVA). n=4 biological donors. (^*^P<0.05, ^**^P<0.001).

Transfection with miR-24 mimic also inhibited IL-6 production, both in positive control and IFN-γ primed cultures, but this effect was lost when IFN-γ priming was performed with the addition of TNF-α, TNF-β, or IL-17A (Figure 3B). Transfection with miR-24 mimic inhibited IL-12 (p40) production under all cultures conditions (Figure 3C). At 96 hours post-stimulation, supernatant levels of IL-10 were significantly lower in miR-24 mimic transfected positive control cultures but not in IFN-γ and IFN-γ and TNF-β primed cultures (Figure 4). There was a trend for increased levels of IL-10 levels in Mφ primed with IFN-γ and TNF-α and IFN-γ and IL-17A, although this was not statistically significant (Figure 4).

### miR-24 overexpression enhances the upregulation of CD206 in alternative but not classically activated Mφ

Mature mD-Mφ were transfected with miR-24 mimic, inhibitor, or negative control mimic, and treated with cytokines that promote classical Mφ activation: IFN-γ and TNF-α, or alternative activation: IL-4 and IL-13 for 72 hours. Cells were analyzed for expression of CD206, CD163, and CD45 by flow cytometry (Table 1). Transfection with miR-24 mimic, but not inhibitor nor control mimic, enhanced IL-4 and IL-13 induced CD206 upregulation. There was a trend for higher CD206 expression in miR-24 mimic transfected cells stimulated with IFN-γ and TNF-α, but was determined to be statistically insignificant. No differences in CD163 or CD45 expression were detected between transfected samples.

### miR-24 overexpression promotes alternative over classical activation under conditions of Mφ plasticity

Mature mD-Mφ were transfected with miR-24 mimic, inhibitor, or negative control mimic, treated with IFN-γ and TNF-α, or IL-4 and IL-13 for 24 hours, at which point media was removed and fresh media supplemented with the opposing cytokines (i.e. IFN-γ and TNF-α treatment was replaced with IL-4 and IL-13, and vice versa). After a further 48 hours of culture, cells were analyzed for expression of CD206, CD163, and CD45 by flow cytometry (Table 1). Transfection with miR-24 mimic, but not inhibitor nor control mimic, diminished the loss of CD206 expression present in cultures that had undergone alternative followed by classical stimulation. No CD206 upregulation was observed under conditions of classical followed by alternative activation and this was not altered by overexpression of miR-24. No differences in CD163 or CD45 expression were detected between transfected samples.

### miR-24 expression decreases PI3 Kinase p110δ expression in Mφ

**Figure 5:**
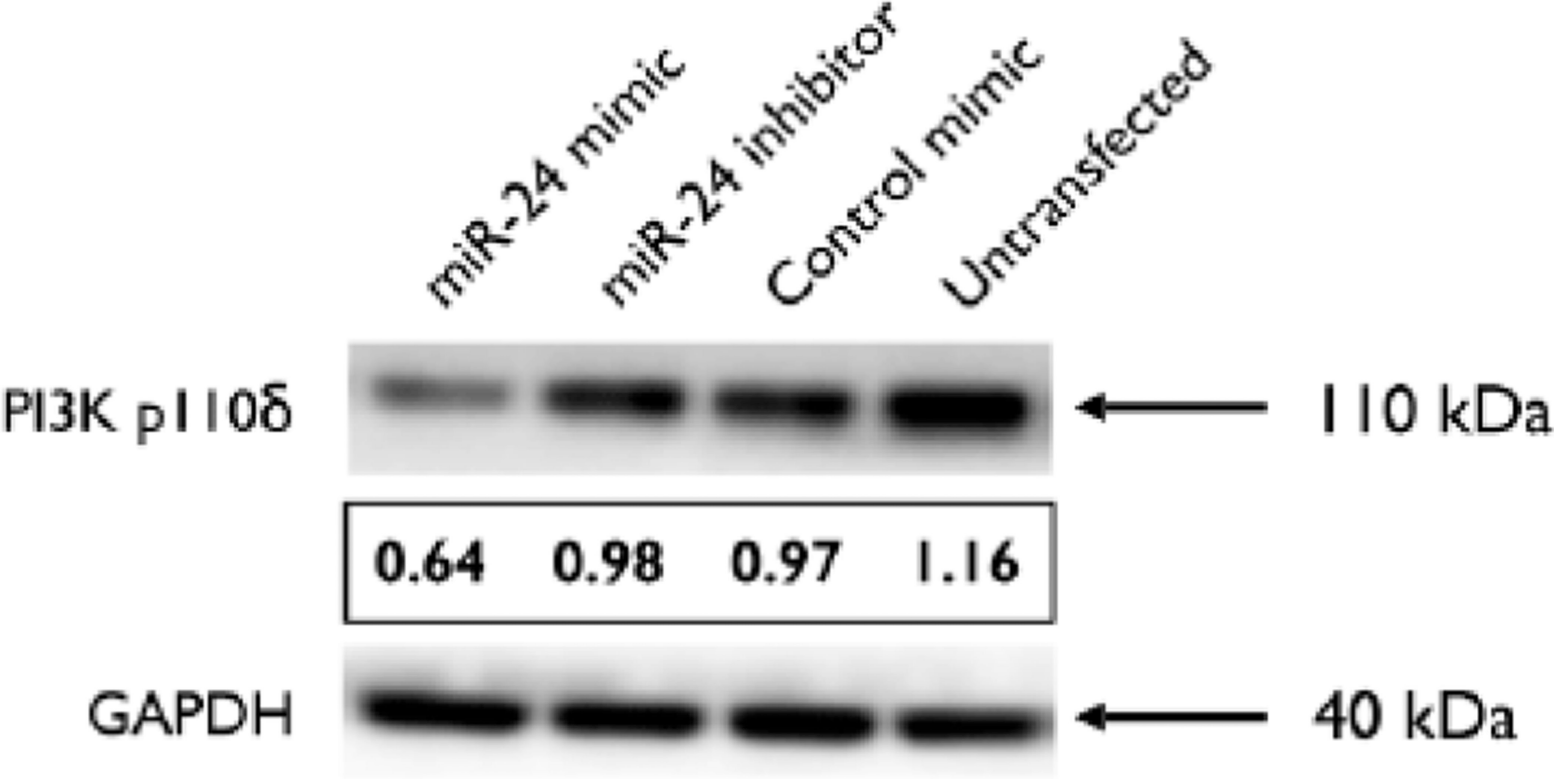
Overexpression of miR-24 in macrophages decreases PI 3-kinase p110δ subunit expression. Macrophages were transfected with miR-24 mimic, inhibitor, control mimic (all at 50 nM), or positive control (untransfected) for 48 h, and expression of p110δ and GAPDH was assessed by western blot analysis. ImageJ software (http://rsbweb.nih.gov/ij/) was used to quantify the results. The values for each lane were normalised with respect to endogenous control GAPDH. Images are representative of 3 biological donors.

PI3 Kinase p110δ was identified as a putative target of miR-24 by *in silico* analysis. Non-primed, non-stimulated mature mD-Mφ were transfected with miR-24 mimic, inhibitor, or negative control mimic and expression of p110δ was assessed by western blot at 36 hours. Transfection of Mφ with miR-24 mimic reduced the relative amount of p110δ protein expression compared to controls (Figure 5).

## Discussion

Activation of Mφ can occur by many means; including the ligation of PRRs by PAMPs (or damage-associated molecular patterns), cytokine-receptor signaling, phagocytosis-associated signaling events and the sensing of endogenous immunomodulatory molecules (such as adenosine and calcium (Ca^2+^)) in the extracellular environment. Previously, we have published data describing miR-24 mediated inhibition of innate Mφ activation; including the reduced phagocytosis of *E. coli* and *S. aureus* bioparticles and associated cytokine production [19], reduced Protein kinase C alpha (PKCα) and NF-κB activation and associated cytokine production [9,19], and the induction of convergent/divergent miRNA expression in Mφ stimulated with Aa-, Pg-, or Pg-CSE-LPS [18]. We now present data describing the regulatory relationship between Mφ, periodontopathic LPS, and miR-24 expression.

An unexpected, although in hindsight predictable, finding was the identification of Mφ activation state as a factor that alters specific parameters of miR-24 mediated inhibition; namely, that classical activation via IFN-γ plus TNF-α, TNF-β, or IL-17A, selectively restores LPS-induced TNF-α secretion to varying degrees. This occurred in the presence of unaltered IL-6 and IL-12(p40) suppression. While it is noteworthy that IFN-γ alone had little impact on restoring TNF-α production, and that a combination of pro-inflammatory cytokines representing the signature cytokines of Th1 (IFN-γ, TNF-α/β) or Th17 (IL-17A, TNF-α/β) cells was required for restoration, it is also difficult to imagine a scenario *in vivo* in which Mφ would receive IFN-γ stimulation in the absence of one of these additional cytokines. Indeed, many of the effects of IFN-γ are associated with a second stimulus, for example IFN-γ priming enhances the subsequent innate activation of Mφ by LPS [22], and classical activation of Mφ is induced by combined IFN-γ-TNF signaling [23]. IL-17 has also been shown to promote classical activation via regulation of NF-κB activity [24,25].

These data also highlight mode of induction dependent differences in what is often described as ‘classical’ Mφ activation- a term that is commonly described as the result of combinations of IFN-γ, TNF-α, and LPS stimulation. LPS stimulation results in direct activation of NF-κB, but also indirect activation via autocrine/paracrine signaling (in which TNF-α is particularly important), while Mφ are able to produce low quantities of IFN-γ (several orders of magnitude lower than that produced by Th1 cells) resulting in TNF-α production via paracrine/autocrine signaling [26]. The importance of these differences is further highlighted by the capacity of structural alterations in LPS to alter cytokine production and miRNA expression, and the observation that different modes of Mφ activation modulate specific miRNA-mediated regulatory effects.

Enhanced expression of the alternative activation marker CD206 in miR-24 overexpressing cells under conditions of alternative but not classical polarization, and during Mφ plasticity from alternative to classical activation states, suggests that miR-24 overexpression does not act simply as a ‘brake’ for classical Mφ activation, but also as an ‘indicator’ signaling intent to move forward on the road towards an alternative phenotype. Within this analogy there are caveats: the fact that a classically activated Mφ is resistant to the miRNA-inhibition of the ubiquitous pro-inflammatory cytokine, TNF-α, suggests that once a Mφ is speeding on the road of classical activation it is more difficult to stop.

The ability of miR-24 to upregulate CD206 expression is indicative of its positive influence on alternative activation. miRNAs are traditionally thought of as negative regulators of gene expression, while classical and alternative Mφ are considered to be opposites due to the fact that their responses and effector functions are tailored towards increased inflammation/tissue destruction and inflammatory catabasis/wound-healing, respectively. Therefore the most likely mechanism/s responsible for the observed phenotype are miR-24 suppression of a negative regulator of alternative activation or a positive regulator of classical activation. Within this dichotomy it is also likely that reduced autocrine/paracrine signaling resulting from suppressed classical/alternative activation will contribute to the increased permissiveness to the opposite activation state. Future work will include looking at direct or indirect regulation of the transcription factors responsible for the balance of classical vs. alternative activation.

Of the cytokines inhibited by miR-24 overexpression, only IL-12(p40) (IL12B) is predicted to be a direct target by *in silico* analysis. Simultaneous inhibition of TNF-α and IL-6 suggests that miR-24 mediated downregulation of IL-12(p40) secretion is more likely to be due to indirect regulation, and reduced p110δ expression may be involved. It is also possible that reduced IL-12(p40) production may be an indirect effect of reduced TNF-α and/or IL-6 autocrine/paracrine signaling. It is also possible that this is mediated/enhanced by reduced receptor expression as miR-24 is predicted to target several cytokine receptors including tumor necrosis factor receptor superfamily (TNFRSF) members 11A (TNFRSF11A), 1B (TNFRSF1B), 10b (TNFRSF10B), 10d (TNFRSF10D), and 19 (TNFRSF19); also interleukin 6 receptor (IL6R), interleukin 12 receptor, beta 1(IL12RB1), and interleukin 10 receptor, beta (IL10RB). It is also possible that miR-24 modulates expression of other PI 3-kinase family members, predicted targets of which include PIK3CG, PIK3C2A, and PIK3C2B. MiR-24 mediated inhibition of IL-10 was only observed in the positive control (no classical activation) group (Figure 4). Levels of IL-10 were also decreased in the classically activated groups (IFN-γ^+/−^ TNF-α/TNF-β/IL-17A) relative to control mimic-transfected Mφ in-line with previous reports of mutual IFN-γ-IL-10 antagonism [27,28], while miR-24 inhibitor had no significant effect on IL-10 levels. The fact that miR-24 overexpression reduces not only early, pro-inflammatory cytokine production (TNF-α, IL-12p40, IL-6) but also late, anti-inflammatory cytokine production (IL-10) raise the possibility that miR-24 is regulating a single signaling molecule that participates in both the rapid and delayed TLR4-induced signaling pathways. To clarify, TLR4 ligation results in the induction of pro-inflammatory cytokines via a MyD88-dependent pathway of NF-κB activation, while the induction of IL-10 by the same stimulus requires the Toll/IL-1R domain-containing adaptor inducing IFN-β (TRIF)-dependent pathway of NF-κB activation- a pathway that is inherently slower due to the involvement of autocrine/paracrine signaling by secondary-response genes, one of which is IL-27 (a predicted target of miR-24) [29]. Further screening of the predicted targets of miR-24 should provide further insight on the miRNA-mediated mechanisms of IL-10 regulation.

With regards to the involvement of p110δ in Mφ activation, it has been reported to be a promoter of TLR-induced cytokine responses, including TLR4. The observation that miR-24 is downregulated by Aa- and Pg-, but not Pg CSE-LPS can be connected, via miR-24 mediated downregulation of p110δ, to the observed reduction in cytokine secretion. Thus one possible mechanism by which cigarette smoke contributes to periodontal pathology is via the modification of LPS resulting in the dysregulation of an endogenous mechanism (miR-24 downregulation) that promotes pro-inflammatory, anti-microbial cytokine secretion.

However, it is noted that while Pg-CSE-LPS induced lower levels of cytokine secretion than Pg-LPS, and miR-24 was downregulated with Pg-LPS stimulation, this hypothesis does not provide an explanation as to why Aa-LPS induced higher levels of cytokine production than Pg-LPS despite a comparable decrease in miR-24 expression. Equally, it does not address the enhancement of IL-4 and IL-13 induced CD206 expression. Explanations include the possibility that this effect is the result of reduced autocrine/paracrine signaling arising from reduced TNF-α/IL-6 secretion, enhanced p110α/β-specific Class IA PI 3 kinase signaling as the result of reduced competition from p110δ for binding to the regulatory subunit, or indeed the regulation and interaction of other miR-24 targets (which is potentially in the hundreds). Investigation of the direct and indirect regulation of the expression of these genes by miR-24 will answer some of the mechanistic questions left standing regarding the observed Mφ phenotype.

miR-24 is highly conserved between species, with identical human (hsa-miR-24-3p) and murine (mmu-miR-24-3p) mature sequence (UGGCUCAGUUCAGCAGGAACAG). Furthermore, our preliminary data from murine bone marrow-derived Mφ (BM-Mφ) demonstrate similar inhibition of LPS-induced cytokine production (data not shown). Thus, future *in vivo* experiments utilizing mouse models of inflammatory disease are of great interest to us. However, current limitations in transfection technology and the relative scarcity/expense of miRNA knock out (KO) mice represent a significant, but not insurmountable, obstacle. In the case of KO mice, many such deletions have proven to be embryonic lethal (revealing the central importance of miRNA regulation for life- and further degrading the notion of miRNAs as merely ‘fine-tuners’ of gene expression). miRNA KOs that are viable still present the same caveats as gene KOs, i.e., elucidation of direct, cell-type/process specific phenotypic effects from those effects that arise indirectly from system-level disturbances; perhaps more so given the fact that a single miRNA may differentially regulate hundreds of genes and that the parameters of this regulation is also dependent upon the differing gene expression profiles of individual cell types. This is further compounded by the rapid, often transient nature of (altered) miRNA expression meaning that the timing of experimentally induced over/underexpression is an important factor in functional outcome. The development of cell-type specific systems of over/underexpression via promoter specificity or delivery systems featuring cell type tropism, as well as recent advancements in inducible systems, such as Tet-On/Tet-Off, should facilitate experiments that deepen our understanding of the role miR-24 plays in immunity.

The results of this study suggest that overexpression of miR-24 in Mφ may be of greater therapeutic benefit for the treatment of inflammatory disorders driven by innate vs. adaptive mechanisms of immunopathology. Insight from *in vivo* data using animal models of inflammatory disease is required to investigate this possibility. Notably, the ability of miR-24 to inhibit Mφ activation, pro-inflammatory cytokine production, and pathogen phagocytosis, may reduce the availability of these signals. This highlights the potential of miR-24 overexpression as a therapeutic for the treatment of inflammatory disease and immunopathological disorders. In summary, this work adds to the body of data describing miR-24 as a negative regulator of the pro-inflammatory, anti-microbial Mφ induced by innate or classical stimuli.

## Figures and Tables

**Table 1:** Overexpression of miR-24 in macrophages enhances CD206 expression during alternative activation and abrogates loss of CD206 expression during the transition from alternative to classical activation. Macrophages were transfected with miR-24 mimic, inhibitor, control mimic (all at 50 nM), or positive control (untransfected) for 18 h, and stimulated with either IFN-γ (10 ng/ml) plus TNF-α (1 ng/ml) or IL-4 (10 ng/ml) plus IL-13 (10 ng/ml) for 48 h; or IFN-γ (10 ng/ml) plus TNF-α (1 ng/ml)/IL-4 (10 ng/ml) plus IL-13 (10 ng/ml) for 24 h followed by replacement of the media with the opposing cytokine combination for a further 48 h, and expression of CD206, CD163 and CD45 was analysed by flow cytometry. Mean values, error bars (standard deviation (SD)), and significance compared to untransfected controls was calculated using Student’s t-test (two-tailed).

	miR-24 mimic			miR-24 inhibitor			Control mimic		
	% positive	SD	P-value	% positive	SD	P-value	% positive	SD	P-value
**Unstimulated**									
CD206	15.1	3.9	NS	18.8	2.7	NS	14.3	4.6	NS
CD163	45.5	9.5	NS	39.5	9.9	NS	37.6	7.7	NS
CD45	96.0	2.3	NS	95.1	2.8	NS	94.9	3.3	NS
**IFN-γ and TNF-α**									
CD206	12.0	2.1	NS	8.7	1.7	NS	4.11	2.9	NS
CD163	7.1	3.5	NS	6.3	4.1	NS	6.6	3.2	NS
CD45	97.2	1.7	NS	96.5	1.6	NS	92.8	4.7	NS
**IL-4 and IL-13**									
CD206	51.2	9.2	*	30.9	6.8	NS	32.7	7.3	NS
CD163	50.4	8.5	NS	52.7	6.2	NS	54.2	7.9	NS
CD45	96.0	1.4	NS	96.2	1.5	NS	96.3	2.8	NS
**IFN-γ and TNF-α>IL-4 and IL-13**									
CD206	14.9	2.3	NS	10.0	1.5	NS	12.6	1.7	NS
CD163	26.8	6.9	NS	33.7	6.1	NS	24.2	8.2	NS
CD45	95.0	1.9	NS	97.5	1.4	NS	92.9	3	NS
**IL-4 and IL-13>IFN-γ and TNF-α**									
CD206	36.8	6.5	*	9.4	2.5	NS	12.8	2.8	NS
CD163	29.7	5.4	NS	25.6	3.4	NS	25.0	8.7	NS
CD45	96.6	1.5	NS	90.5	2.0	NS	94.0	1.8	NS

n=4 biological donors. (*P<0.05).

NS=not significant.
